# Mutations in the plastidic ACCase gene endowing resistance to ACCase-inhibiting herbicide in *Phalaris minor* populations from India

**DOI:** 10.1007/s13205-015-0331-4

**Published:** 2016-01-05

**Authors:** Nishu Raghav, Rajender Singh, Rajender Singh Chhokar, Davinder Sharma, Raman Kumar

**Affiliations:** 1 ICAR-Indian Institute of Wheat and Barley Research, Post Box 158, Karnal, 132001 India; 2 Maharishi Markandeshwar University, Mullana, India

**Keywords:** Acetyl-coenzyme A carboxylase, Herbicide resistance, Single nucleotide polymorphism, Mutation, *Phalaris minor*

## Abstract

Littleseed canarygrass (*Phalaris minor* Retz.) is one of the most common and troublesome weeds infesting wheat crop in India. Repeated use during the last two decades of the ACCase-inhibiting herbicide (clodinafop) to control this weed has resulted in the occurrence of resistance. Fifty-three *P. minor* populations were collected from wheat fields in Haryana and Punjab states of India. The dose–response assays indicated that 29 populations were resistant, 23 populations were susceptible and one population was moderately resistant to clodinafop. Sequence analysis of the CT domain of ACCase gene among resistant and susceptible populations revealed two non-synonymous mutations, Trp_2027_ to Cys and Ile_2041_ to Asn in the resistant populations. Allele-specific PCR markers were developed to differentiate between wild-type and resistant codons at positions 2027 and 2041 of ACCase in *P. minor* which enables molecular assays for rapid detection and resistance diagnosis for efficient weed management in wheat. This is the first report from India of a target site mutation corresponding to resistance to clodinafop in *P. minor.*

## Introduction

Littleseed canarygrass (*Phalaris minor* Retz.) is the most dominant winter season weed in wheat and widely distributed in wheat growing regions of the world. This weed has been reported from Macronesia to Mediterranean, Irano-Turanic and Saharo-Sindic regions, eastern and South Africa, North and South America, Australia and the Far East (Singh et al. 1999). In India, it is the major weed of the north-western and eastern Indo-Gangetic Plains (Chhokar and Sharma 2008). Up until the early 1990s, *P. minor* was effectively controlled by isoproturon, a substituted urea herbicide, but after long period of continuous and heavy spraying along with poor application rates, spray techniques and timing led to the evolution of isoproturon resistance in *P. minor* (Malik and Singh 1995; Chhokar and Malik 2002). In 1997–1978, four alternative herbicides (sulfosulfuron, clodinafop, fenoxaprop and tralkoxydim) were recommended for its control and the majority of farmers accepted clodinafop due to excellent crop safety and efficacy against this weed. The excessive dependence on clodinafop resulted in evolution of weed resistance against this herbicide. At present, this weed has evolved multiple herbicide resistance across three modes of actions: photosynthesis at the photosystem II site A, acetyl CoA carboxylase and acetolactate synthase inhibition (Chhokar and Sharma 2008). Herbicide resistance in weeds is an evolutionary process and its dynamics and impact are dependent on several factors such as genetics and biology of weeds along with herbicidal, operational and other biological components (Powels and Yu 2010). There are currently 418 unique cases (species x site of action) of herbicide-resistant weeds globally, with 224 species (129 dicots and 95 monocots). Weeds have evolved resistance to 21 of the 25 known herbicide sites of action and to 149 different herbicides. Herbicide-resistant weeds have been reported in 73 crops in 61 countries (Heap 2014).

Aryloxyphenoxypropionate (APP) and cyclohexanedione (CHD) herbicides inhibit plastid form of acetyl-CoA carboxylase by blocking the fatty acid biosynthesis which results in disruption of cell membrane integrity and consequently causing metabolite leakage and rapid plant death (Devine and Shimabukuro 1994). Acetyl-coenzyme A carboxylase (ACCase) (EC.6.4.1.2) is a key enzyme in fatty acid biosynthesis catalyzing ATP-dependent carboxylation of acetyl-CoA to malonyl-CoA. All ACCase isoforms contain three distinct functional domains: the biotin carboxyl carrier (BCC) as structural domain, and the biotin carboxylase (BC) and the carboxyl transferase (CT) domains as catalytic domains (Nikolau et al. 2003). The ACCase enzyme activity is inhibited by APP and CHD herbicides by binding within the CT domain of the plastidic ACCase in susceptible plant species and thus the CT domain of the ACCase gene determines the herbicide sensitivity in plants (Nikolskaya et al. 1999). Several point mutations have been reported in the CT domain of plastid-localized ACCase responsible for the enzyme insensitivity to herbicides in several grass weeds (reviewed by Kukorelli et al. 2013; Beckie and Tardif 2012; Powles and Yu 2010). Therefore, the search for mutations endowing ACCase herbicide resistance was conducted by sequencing the CT domain of the plastidic ACCase gene in *P. minor*. In this study, we elucidated that the amino acid substitutions in CT domain of plastidic ACCase gene are associated with herbicide resistance in *P. minor*. Allele-specific PCR-based test further confirmed the differentiation of herbicide-resistant and susceptible populations.

## Materials and methods

### Plant material

Seeds of 53 *P. minor* populations were collected during April 2013 from the wheat fields in Haryana and Punjab states of India. One population (G-46), an identified susceptible check for clodinafop, was also included in the study. Pot studies were conducted to determine the response of various populations to clodinafop. The pots were filled with soil from a field having previously no infestation of *P. minor* and well-rotten farm yard manure in 6:1 ratio (v/v) after passing through 2 mm sieve. The pots were frequently irrigated to deplete the soil seed bank, if any before sowing. The seedlings were established by seeding 50–60 seeds at a depth of 2.5 cm. After 2 weeks of emergence, thinning was done to maintain 15 plants per pot. The herbicide was sprayed at 3 leaf stages of *P. minor* with Knapsack sprayer fitted with flat fan nozzles. Herbicide treatments were clodinafop (Topik 15 WP; Syngenta India Ltd., Mumbai, India) at 0, 30 and 60 g/ha. Four weeks after spraying, the herbicide response was recorded and leaves of different populations were taken for DNA extraction. The leaf sample for DNA extraction were taken from the control (unsprayed) pots for the susceptible biotypes effectively controlled at ½ X dose (0.30 g/ha), whereas the leaf samples of resistant biotypes were taken from plants survived after spray of X (0.60 g/ha) dose of herbicide.

### DNA extraction and plastidic ACCase CT domain sequencing

Genomic DNA was extracted from seedlings using CTAB method (Doyle and Doyle 1990). A polymerase chain reaction (PCR) was performed in a 25-μL volume containing 100 ng of genomic DNA, 2.5 μL of 10× *Taq* PCR buffer, 1.5 mM of MgCl2, 200 μM of each dNTP, 0.2 μM of each primer and 1.0 unit of *Taq* DNA polymerase. The thermocycling program consisted of an initial denaturation at 94 °C for 4 min, followed by 30 cycles of 45 s at 94 °C, 30 s at 60 °C, 60 s at 72 °C and a final cycle of 5 min at 72 °C in S 1000 Thermal Cycler (Bio-Rad). The amplified product was separated by electrophoresis on 2 % (w/v) agarose gel and stained with ethidium bromide and analyzed under UV light.

The amplicons were purified using HiPurATM agarose gel DNA purification spin kit (HiMedia Laboratories Pvt. Ltd., Mumbai, India) according to the manufacturer’s instructions and sequenced with primers used for amplification from both the directions to minimize the false SNP due to sequencing artifacts. The sequencing was performed by Eurofins Genomics India Pvt. Ltd, Banglore, India). The sequence alignments were done using ClustalW2 (http://www.ebi.ac.uk/Tools/msa/clustalw2/) with default parameters and allele-specific primers were designed for SNPs (Table 1).

**Table 1:** Primers used for sequencing and allele-specific PCR analysis of mutations

Primer	Sequence (5′–3′)	*T*_m_ (°C)	Experiment	References
Up ACCase-B	AAGGATGGGCGAAGACAGTAGTTA	62	Sequencing	Gherekhloo et al. (2012)
Low ACCase-B	CTCCATCAGATAGGCTTCCATTT			
PM2027F	TTCCCATGAGCGGTCTGTTC	55	Allele-specific PCR for 2027	This study
PM2027R	GCCCACCAGAGAAGCCTCT**A**			
PM2041F	CATGATGCAGCTTGTCCCTG	63	Allele-specific PCR for 2041	This study
PM2041R	TTGACCCAGCCTGCAGA**T**			

Bold and underlined nucleotides in primers represent the nucleotide substitution in herbicide-resistant biotypes

## Results and discussion

The variable crop phytotoxicity to clodinafop treatments were observed in the weed populations. The resistant populations were not affected by recommended dose (X dose) of clodinafop (60 g/ha), whereas susceptible populations were effectively controlled by ½ X dose (30 g/ha) of clodinafop (Table 2). The dose–response assays indicated that 29 populations were resistant, 23 populations were susceptible and one population was moderately resistant to clodinafop.

**Table 2:** Response of various populations of *P. minor* to clodinafop

Visual phytotoxicity (%)	Clodinafop (30 g/ha)	Clodinafop (60 g/ha)
0–25	–	H-14, H-15, H-16, H-17, H-19, H-20, H-21, H-22, H-26, H-28, H-31, H-34, H-40, P-5, P-12, P-17, P-38, P-49, P-54, P-75, P-76, G-8, G-21, G-29, G-34, G-43, N-5, N-8, N-9
26–50	–	H-13
51–75	–	–
76–100	H-8, H-23, H-27, H-68, P-8, P13, P-14, P-26, P-30, P-35, P-43, P-60, P-63, P-69, G-2, G-6, G-18, G-39, G-40, G-42, G-46, G-47, N-4	–

**Fig. 1:**
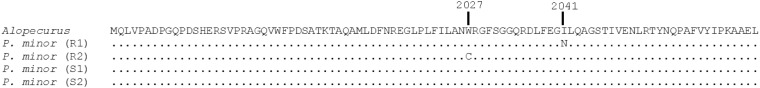
Amino acid sequence comparisons of herbicide target site in the CT domain of ACCase gene. Mutations associated with resistance are shown with residue numbering following the full-length *A. myosuroides* plastid ACCase (GenBank accession No. AJ310767). The substitution found in the two resistant populations of *Phalaris minor* (R1 and R2) in comparison with two susceptible populations (S1 and S2) is highlighted at amino acid position at 2027 and 2041

To identify the molecular basis of resistance, the CT domain of ACCase gene from five individuals of two resistant and two susceptible populations was sequenced (GenBank accession No. KF770792, KF770793, KF770794 and KF770795). The sequences were aligned with CT domain of plastidic ACCase genes of *Alopecurus myosuroides* (GenBank accession No. AJ310767). Two non-synonymous mutations were found in the resistant and susceptible biotypes of *P. minor* (Fig. 1). The nucleotide sequences of one resistant populations differed from susceptible by a single nucleotide substitution of G/T that resulted in substitution of Trp_2027_ to Cys in the resistant population, whereas a substitution of T/A resulted in substitution of Ile_2041_ to Asn (numbered according to the *Alopecurus myosuroides* plastid ACCase) in another resistant population.

**Fig. 2:**
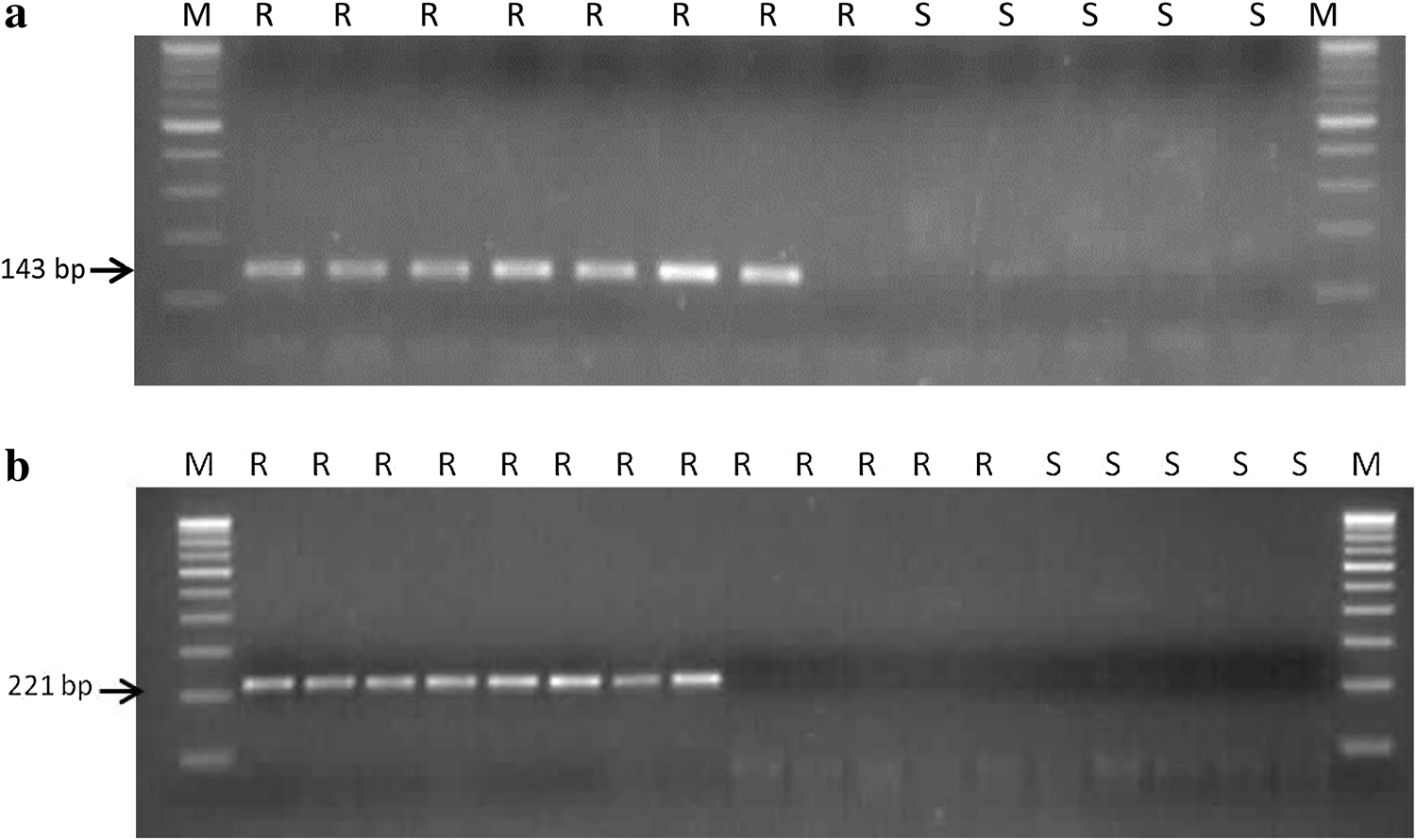
Allele-specific PCR for ACCase mutations, **a** Trp_2027_ to Cys and **b** Ile_2041_ to Asn in herbicide-resistant populations of *P. minor.* *M* 100 bp DNA ladder, *R* resistant population, *S* susceptible population

Based on the sequence analysis results for the mutation site, allele-specific PCR was designed from the position of base substitution to further characterize resistant and susceptible populations (Table 1). The rationale in designing the primers was based on the premise that the 3′-terminal positions ought to be unique for the herbicide-resistant biotype. The allele-specific primers were tested on 53 populations which were phenotyped for herbicide resistance. PCR fragment of 143 bp size was obtained from seven resistant populations with primer pair PM2027F/PM2027R associated with Trp_2027_ to Cys mutation, whereas no PCR product was amplified in susceptible populations (Fig. 2a). Similarly, a 221 bp DNA fragment was amplified from 15 resistant populations associated with Ile_2041_ to Asn mutations with PM2041F/PM2014R primer pair (Fig. 2b). Two populations (P49 and P54) found to possess both the point mutations based on PCR products. However, the remaining 10 resistant populations were not found to endow these point mutations associated with clodinafop resistance and may possess other mutations responsible for herbicide resistance.

Twelve target site mutations (amino acid substitutions) in the ACCase gene have been documented till date in grass weed species (Beckie and Tardif 2012; Kaundun et al. 2013). The Trp_2027_ to Cys substitution has also been reported in Iranian *P. minor* populations to endow resistance to ACCase inhibitors (Gherekhloo et al. 2012) and the Ile_2041_ to Asn mutation has been reported in awned canary-grass (*Phalaris paradoxa*) in Israel (Hochberg et al. 2009). The Trp_2027_Cys and Ile_2041_Asn mutations confer resistance to APPs (fenoxaprop, clodinafop, haloxyfop). The Trp_2027_Cys mutation also confers resistance to pinoxaden, whereas Ile_2041_Asn pinoxaden may confer moderate resistance to pinoxaden. The both mutations were not found associated with resistance to CHDs (Petit et al. 2009; Scarabel et al. 2011). The experiments with yeast gene-replacement strains confirmed that single amino acid changes in Trp_2027_–Cys and Ile_2041_–Asn confer strong resistance to APPs and mild resistance to CHDs (Liu et al. 2007). The three-dimensional model of CT-herbicide complex in black-grass revealed that the Ile_2041_ to Asn substitution interferes with herbicide binding because of change in APP binding site (Délye et al. 2005). The Trp_2027_ to Cys substitution did not interfere with APP binding but resulted in small allosteric changes which might hamper the access of herbicide to the binding site (Délye et al. 2005). Another computational study on Trp_2027_ to Cys, Ile_2041_ to Asn, Asp_2078_ to Gly, and Gly_2096_ to Ala mutations suggested a large effect on conformation of the binding pocket and the hydrogen bonding interactions. The mutant-type ACCase has a lower affinity for inhibitor binding than the wild-type enzyme, which account for the molecular basis of herbicide resistance (Zhu et al. 2009).

Resistance to herbicides in weeds is increasing rapidly worldwide and threatening global food security. Resistance has now been reported to all major herbicide modes of action despite the development of resistance management strategies in the 1990s. Herbicide resistance is now widely recognized as the result of the adaptive evolution of weed populations to the intensive selection pressure exerted by herbicides. The least herbicide-sensitive individuals repeatedly sprayed with herbicide have a selective advantage in the weed populations and thus increase in frequency until populations shift towards a predominance of herbicide-resistant individuals (Délye et al. 2013). With patents expiring on many ACC inhibitors worldwide, greater usage of these herbicides as a consequence of falling prices is to be expected in the future and the projected usage trend will be countered by ever-increasing incidence of resistance to herbicides (Beckie and Tardif 2012). Therefore, an efficient and rapid diagnosis of resistance is essential to maintain herbicide efficacy by preventing further resistance selection when resistance has evolved in a weed population and to avoid ineffective herbicide applications.

## Conclusion

The continuous adoption of rice–wheat cropping system in wheat growing regions has led to the problem of *Phalaris minor* as a troublesome weed of wheat causing yield reductions. Reliable, fast, simple detection of herbicide resistance is necessary for farmers to adopt timely alternative herbicide application strategies to prevent further spread of herbicide-resistant weeds. DNA-based diagnostic markers have the advantage of quickly detecting resistant plants compared with conducting pot assays, which are time and space consuming, and labor intensive. The primary aim of the study was to find the mutations in CT domain of plastidic ACCase gene conferring herbicide resistance in *P. minor*. The allele-specific PCR marker developed based on SNP will help in identification clodinafop-resistant and susceptible populations.

## References

[CR1] Beckie HJ, Tardif FJ (2012). Herbicide cross resistance in weeds. Crop Prot.

[CR2] Chhokar RS, Malik RK (2002). Isoproturon resistant *Phalaris minor* and its response to alternate herbicides. Weed Technol.

[CR3] Chhokar RS, Sharma RK (2008). Multiple herbicide resistance in littleseed canarygrass (*Phalaris minor*): a threat to wheat production in India. Weed Biol Manag.

[CR4] Délye C, Zhang X-Q, Michel S, Matéjicek A, Powles SB (2005). Molecular basis for sensitivity to acetyl-conzyme A carboxylase inhibitors in black-grass. Plant Physiol.

[CR5] Délye C, Jasieniuk M, Le Corre V (2013). Deciphering the evolution of herbicide resistance in weeds. Trends Genet.

[CR6] Devine MD, Shimabukuro RH, Powles SB, Holtum JAM (1994). Resistance to acetyl coenzyme A carboxylase inhibiting herbicides. Herbicide resistance in plants.

[CR7] Doyle JJ, Doyle JL (1990). Isolation of plant DNA from fresh tissue. Focus.

[CR8] Gherekhloo J, Osuna MD, De Prado R (2012). Biochemical and molecular basis of resistance to ACCase-inhibiting herbicides in Iranian *Phalaris minor* populations. Weed Res.

[CR9] Heap I (2014) 2014 The international survey of herbicide resistant weeds. http://www.weedscience.org. Accessed 2 Jan 2014

[CR10] Hochberg O, Sibony M, Rubin B (2009). The response of ACCase-resistant *Phalaris paradoxa* populations involves two different target site mutations. Weed Res.

[CR11] Kaundun SS, Bailly GC, Dale RP, Hutchings S-J, McIndoe E (2013). A novel W1999S mutation and non-target site resistance impact on acetyl-CoA carboxylase inhibiting herbicides to varying degrees in a UK *Lolium multiflorum* population. PLoS One.

[CR12] Kukorelli G, Reisinger P, Pinke G (2013). ACCase inhibitor herbicides-selectivity, weed resistance and fitness cost: a review. Int J Pest Manag.

[CR13] Liu W, Harrison DK, Chalupska D, Gornicki P, O’Donnell CC, Adkins SW, Haselkorn R, Williams RR (2007). Single-site mutations in the carboxyltransferase domain of plastid acetyl-CoA carboxylase confer resistance to grass-specific herbicides. Proc Natl Acad Sci.

[CR14] Malik RK, Singh S (1995). Littleseed canarygrass (*Phalaris minor* Retz.) resistance to isoproturon in India. Weed Technol.

[CR15] Nikolau BJ, Ohlrogge JB, Wurtele ES (2003). Plant biotin-containing carboxylases. Arch Biochem Biophys.

[CR16] Nikolskaya T, Zagnikto O, Tevzadze G, Haselkorn R, Gornicki P (1999). Herbicide sensitivity determinant of wheat plastid acetyl-CoA carboxylase is located in a 400-amino acid fragment of the carboxyltransferase domain. Proc Natl Acad Sci.

[CR17] Petit C, Bay G, Pernin F, Délye C (2009). Prevalence of cross- or multiple resistance to the acetyl-coenzyme A carboxylase inhibitors fenoxaprop, clodinafop and pinoxaden in black-grass (*Alopecurus myosuroides* Huds.) in France. Pest Manag Sci.

[CR18] Powles SB, Yu Q (2010). Evolution in action: plants resistant to herbicides. Annu Rev Plant Biol.

[CR19] Scarabel L, Panozzo S, Varotto S, Sattin M (2011). Allelic variation of the ACCase gene and response to ACCase-inhibiting herbicides in pinoxaden-resistant *Lolium* spp. Pest Manag Sci.

[CR20] Singh S, Kirkwood RC, Marshall G (1999). Biology and control of *Phalaris minor* Ritz. (littleseed canarygrass) in wheat. Crop Prot.

[CR21] Zhu X-L, Ge-Fei H, Zhan C-G, Yang G-F (2009). Computational simulations of the interactions between acetyl-coenzyme A carboxylase and clodinafop: resistance mechanism due to active and nonactive site mutations. J Chem Inf Model.

